# Design of an Edge-Detection CMOS Image Sensor with Built-in Mask Circuits

**DOI:** 10.3390/s20133649

**Published:** 2020-06-29

**Authors:** Minhyun Jin, Hyeonseob Noh, Minkyu Song, Soo Youn Kim

**Affiliations:** Department of Semiconductor Science, Dongguk University, Seoul 04620, Korea; mhjin91@dongguk.edu (M.J.); nhs_0410@naver.com (H.N.); mksong@dongguk.edu (M.S.)

**Keywords:** CMOS image sensor, computer vision, edge detection, low power consumption, single-slope ADC

## Abstract

In this paper, we propose a complementary metal-oxide-semiconductor (CMOS) image sensor (CIS) that has built-in mask circuits to selectively capture either edge-detection images or normal 8-bit images for low-power computer vision applications. To detect the edges of images in the CIS, neighboring column data are compared in in-column memories after column-parallel analog-to-digital conversion with the proposed mask. The proposed built-in mask circuits are implemented in the CIS without a complex image signal processer to obtain edge images with high speed and low power consumption. According to the measurement results, edge images were successfully obtained with a maximum frame rate of 60 fps. A prototype sensor with 1920 × 1440 resolution was fabricated with a 90-nm 1-poly 5-metal CIS process. The area of the 4-shared 4T-active pixel sensor was 1.4 × 1.4 µm^2^, and the chip size was 5.15 × 5.15 mm^2^. The total power consumption was 9.4 mW at 60 fps with supply voltages of 3.3 V (analog), 2.8 V (pixel), and 1.2 V (digital).

## 1. Introduction

In recent years, complementary metal-oxide-semiconductor (CMOS) image sensors (CIS) with computing functions (called computer vision sensors) have received considerable attention for use in a wide variety of applications, such as medical imaging, automotive safety, surveillance, and face detection for auto-focus in digital cameras [1,2,3,4,5,6,7]. For edge/face/object detection processing in mobile applications that are battery powered, low power consumption is a primary design criterion. In essence, computer vision (CV) sensors capture, process, analyze, and understand the objects [8,9,10,11]. To detect objects using CV computation, two types of approaches have been developed: (1) asynchronous and event-based sensors, such as dynamic vision sensors (DVS), and (2) frame-based CIS with CV computation hardware.

Event-based sensors, such as DVS [12], asynchronous time-based image sensors [10], and dynamic and active-pixel vision sensors (DAVIS) [11], have been developed for low-latency CV computation. DVS asynchronously detects pixel-level temporal differences in images, called an “event” within a microsecond of time, and transmits the output to track fast-moving objects [13,14]. Furthermore, since no information is transformed without events, power consumption at the system level can be saved and reduced by a factor of 100 compared with the frame-based CIS with CV hardware. However, DVS only informs 1-bit resolution output that has an x, y-address in the pixel array in which temporal contrast changes. Hence, the outputs of DVS cannot be adapted for traditional CV algorithms that require static scene information rather than dynamic event information. As another type of event-based sensor, DAVIS combines the advantages of the two types of sensors: DVS and frame-based CIS. However, while DVS tracks the dynamic events of temporal difference, frame-based CIS captures static scene information to apply traditional CV algorithms with additional CV hardware, leading to high power consumption and high latency. In addition, a pixel of asynchronous sensors consists of a photodiode, 47 transistors, and 2 capacitors, while traditional active pixel sensors (APS) have 4 transistors (4-T) and a photodiode only. Therefore, the fill factor (ratio of pixel array’s light-sensitive area to its total area) of DVS is about 20~30%, so there is a limitation in achieving high image resolution sensors (~magnitude of megapixels). 

On the other hand, framed-based CIS has been used with CV computation hardware in a range of applications [8,9]. In frame-based CIS, the analog information in voltage acquired from conventional 4-T APS is converted to the digital domain using an analog-to-digital converter (ADC). The digitized outputs are sent to an image signal processor (ISP) that performs certain CV operations to analyze and understand the information received from the individual pixels to detect features such as faces, pedestrians, and gestures. Owing to the mature development of APS and ADC, high-performance and high-resolution frame-based CIS can be used for further CV applications, such as long-distance object detection for surveillance sensing systems. However, reading out the image information frame by frame causes frame-based CIS to suffer from a long data-processing time as image resolution increases [15,16]. In addition, since CV processing to detect face or other objects requires significant power consumption, this approach cannot be readily applied to mobile applications because of its lower battery life. 

Therefore, for power-efficient CV operations, by taking advantage of mature APS and ADC, simple CV computation hardware for edge detection can be implemented in column-parallel peripheral circuitry based on a frame-based CIS structure. In this paper, we propose a power-efficient CIS with a built-in mask that outputs edge images without an additional ISP by using the conventional CIS structure. In addition, the proposed CIS can selectively capture either edge-detection images or normal 8-bit images that enable static scene information to be used for existing CV algorithms. The contents of this paper are as follows. Section 2 discusses the proposed edge-detection CIS alongside its circuit design and implementation. The experimental results and conclusions are summarized in Section 3 and Section 4, respectively.

## 2. Design of the Proposed Edge-Detection CMOS Image Sensor 

### 2.1. Existing Edge-Detection Mask Algorithm

**Figure 1:**
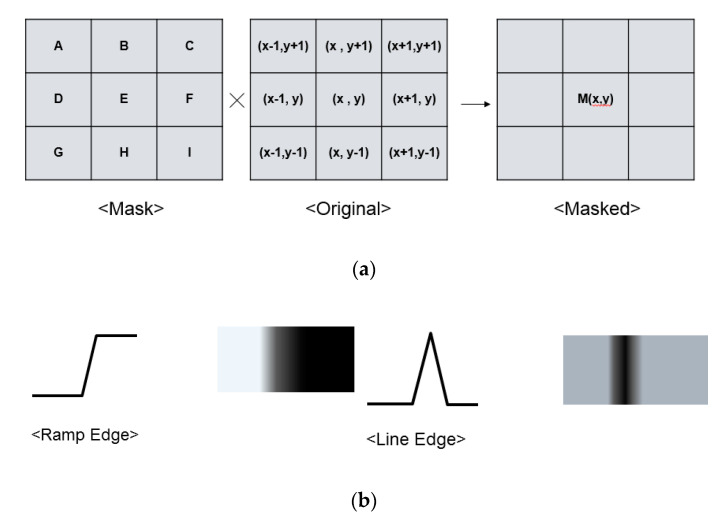
(**a**) Principle of the mask technique and (**b**) different types of edges.

To detect edges, the mask-processing technique is generally used. Using the mask, the edges are recognized as a valid value, and the rest of the image data are zeroed. Because the edges are always constant regardless of the illuminance, edge images are independent of the measurement environment. Figure 1a shows the principle of the mask-processing technique for edge detection. When a 3 × 3 mask is overlaid on the original target image, each cell of the mask is applied to eight pixels around the center pixel (x, y). The cells A–I of the mask each give a constant weight to the coordinates of the image pixel. The center pixel (x, y) is multiplied by the weight of each mask cell and the pixel value and then added together to change to a new M (x, y). This process is applied to all cells that correspond to the image resolution to form a new image. In different types of mask techniques, such as Sobel, Canny, Prewitt, Roberts, Laplacian, and Gaussian, edges can be obtained with different weights [17,18]. As shown in Figure 1b, there are different types of edges: a ramp edge and a line edge. The ramp edge changes the brightness of the pixel after it rapidly changes brightness. On the other hand, the line edge changes instantaneously and then returns to the initial value. In reality, most of the edges are in the form of ramp edges, so converting them to line edges is the principle of edge detection [17]. As can be seen from the waveforms of the two edges in Figure 1b, the change from the ramp edge to the line edge is equivalent to differentiating the ramp edge waveform. That is, by detecting when there is a sudden change in image pixel data, an edge of the image can be detected. The basic shapes of different types of masks, such as Sobel, Canny, Prewitt, and Roberts, are the same. The larger the data change from the center pixel to the nearby pixel, the more the M (x, y) value is increased. When the calculated value exceeds a specific threshold, M (x, y) is defined as edge data, resulting in ‘1’. On the other hand, if M (x, y) is less than the threshold, it is defined as ‘0’. Since these techniques detect horizontal and vertical edges separately, two masks for horizontal and vertical edges are required. After processing horizontal and vertical edges, the result of the two masks is added through OR logic. However, such existing mask techniques simultaneously require three columns and three rows for one mask process. To implement these existing mask techniques, edge-detection mask processing is performed in an additional chip called an ISP, which communicates with a conventional CIS chip. In addition, conventional CISs read out the pixel data row by row, and a row buffer to store the pixel data of these three rows is required, increasing the overall area and power consumption [7]. In this case, the additional row buffer increases power consumption and requires high-speed operation because of the additional operation of the row buffer consisting of additional memory blocks. 

To implement the edge-detection process in a conventional CIS, a row buffer with several rows has been used to design a 3 × 3 mask circuit in a circuit, which greatly increased the layout size and power consumption of the entire block [5]. Additionally, a global shutter method through an in-pixel ADC has been used [6], which requires a different type of pixel design. This in-pixel ADC decreases the pixel’s fill factor, thus greatly increasing the unit cell size of the pixel. In turn, this limitation prevents high resolution from being achieved. Another approach can detect fixed pixel values of edges for iris recognition without using a mask algorithm [7]. However, the data value of the iris is assumed to range from 120 to 180 on the basis of 8-bit 256 codes, so obtaining edge images can vary depending on the illuminance. To create a power-efficient edge-detection CIS, we propose a new mask technique that can be implemented in a CIS’s column-parallel ADC without a row buffer while minimalizing the conventional CIS structure. In addition, the proposed mask circuit enables the CIS to obtain edge images regardless of the illuminance with low power consumption. 

### 2.2. Proposed Algorithm for Edge Detection

**Figure 2:**
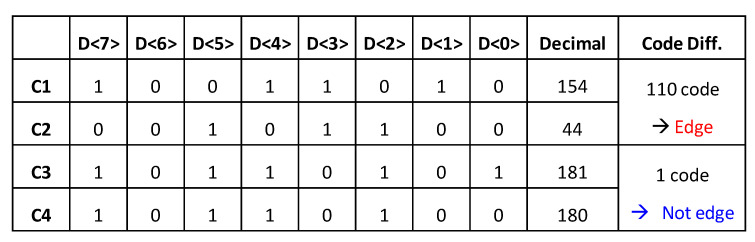
Edge-detection process during readouts in CMOS image sensors (CISs).

**Figure 3:**
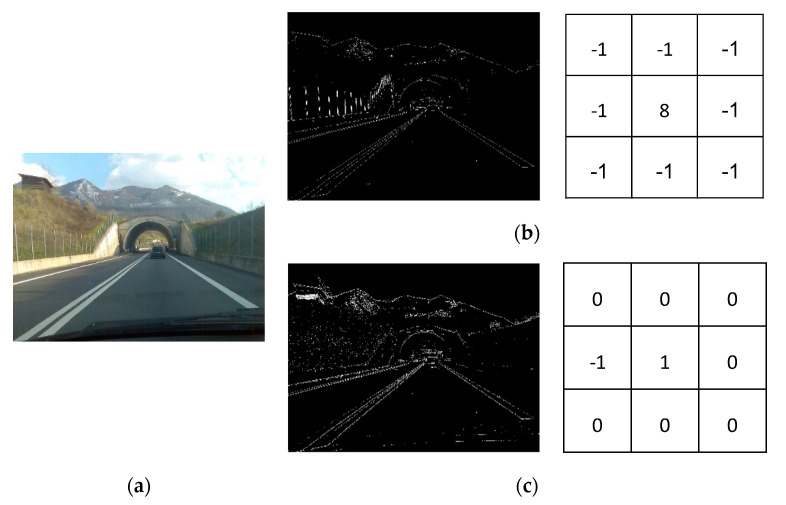
Comparison of the Laplacian and proposed masks: (**a**) an original image and an edge image that uses (**b**) a Laplacian mask (for eight neighboring pixels) and (**c**) the proposed mask.

The proposed edge-detection algorithm uses the read-out process of the proposed CIS, in which all column data are sequentially read as 8-bit signals. The read-out operation of a conventional CIS is as follows. First, when the pixels of one row are selected, all the column data of the row are stored in static random-access memory (SRAM) by each column ADC and then read out sequentially from the first column. In this process, the difference in the output code of data between adjacent columns that are stored in SRAM can be obtained to detect edges. Figure 2 shows an example of edge detection during the read-out process. When the threshold value of the code difference between two columns that are judged as an edge is 10, 110 code changes are present from 001,011,00_2_ (C1) to 100,110,10_2_ (C2), resulting in an edge. On the other hand, if one code change exists from 100,110,10_2_ (C3) to 001,011,00_2_ (C4), the region is not an edge because one is less than the threshold of 10. The proposed algorithm can perform functions such as a vertical edge-detection mask because the data-output code difference between columns is detected as an edge. Figure 3 shows a comparison of the edge images and mask for a Laplacian mask and the proposed mask. The edge-detection algorithm recognizes only the edge of the entire image as a valid value and sets all the remaining data to zero. In particular, the road has a diagonal shape, so the shape of the road is accurately recognized even by edge detection between vertical directions. In addition, by detecting only one vertical edge, the result shows that the proposed mask is less sensitive to noise compared with the Laplacian mask. Additionally, the proposed mask can be easily implemented in the conventional CIS structure because of the mask’s simplicity, and the proposed edge-detection CIS can selectively capture either edge-detection 1-bit images or normal 8-bit images. 

### 2.3. Operation Principle of the Edge-Detection CMOS Image Sensor 

**Figure 4:**
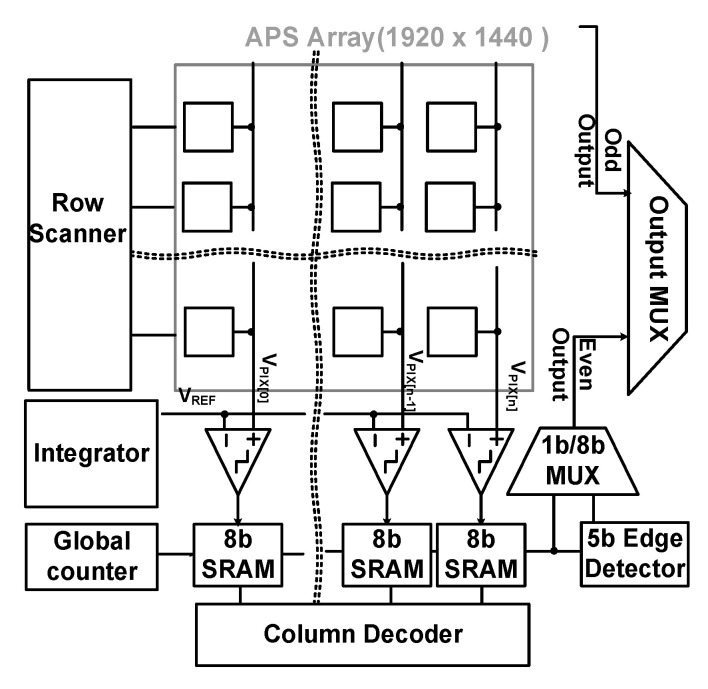
Block diagram of the proposed edge-detection CIS.

**Figure 5:**
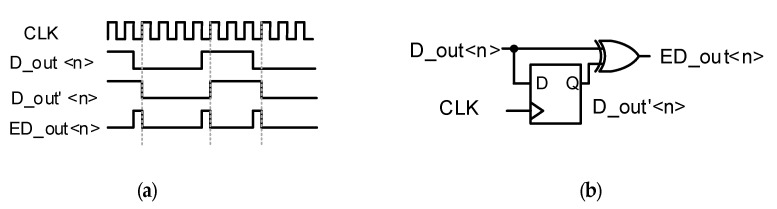
(**a**) Timing diagram and (**b**) circuit of the edge detector.

Figure 4 shows a block diagram of the proposed edge-detection CIS when using an 8-bit single-slope ADC (SS-ADC). The SS-ADC has been generally used in CIS because of the small area and simple structure [19,20,21,22,23,24,25,26]. The resolution of the CIS is 1920 × 1440 with a 4-shared APS array. Unlike conventional CISs, an edge detector is implemented by using a flip-flop and an exclusive-OR (XOR) gate. An input signal that is received by a pixel is processed by one row through a rolling-shutter method, and a digitally coded multi-bit signal is stored in each column’s 8-bit SRAM array. When processing one row, 8-bit signals of each column in the 8-bit SRAM are output as a series of digital pulse signals, D_out<n>, as shown in Figure 5. 

The edge-detector circuit can detect the positive and negative edges of D_out<n> and can detect the change in each bit output from the SRAM. The bit of the edge detector is determined according to the edge threshold. The difference between adjacent pixels that exceed the 15 least significant bits (LSB) out of 256 full-scale codes is less than 10% of the entire image, and this value decreases as the image resolution increases [27]. Therefore, if the difference is more than 8 LSB in the image, the change could be considered sufficiently sudden, and the region could be processed as an edge in the image. Therefore, the five most significant bits (MSB) of the 8-bit SRAM output go to 5-bit signal edge detectors because the edge threshold is 8 LSB by default. The edge threshold is selectable among 8, 16, and 32. However, in some cases, the value of the most significant 6 bits may be changed by a carry that is caused by a lower bit, for example, from 000,001,11_2_ to 00001000_2_. To avoid this case, we designed an error-correction logic consisting of multiplexers (MUX) by adding an offset to the n + 1 column. For example, when the MSB of 3-bit SRAM (not used for edge detection) is ‘H’, the carry generation signal controls a mux to select the Qb instead of Q, leading to a ‘−1’ code offset of the LSB of 5-bit static random access memory (SRAM) [28].

## 3. Experimental Results

### 3.1. Simulation Results and a Chip Photograph

**Figure 6:**
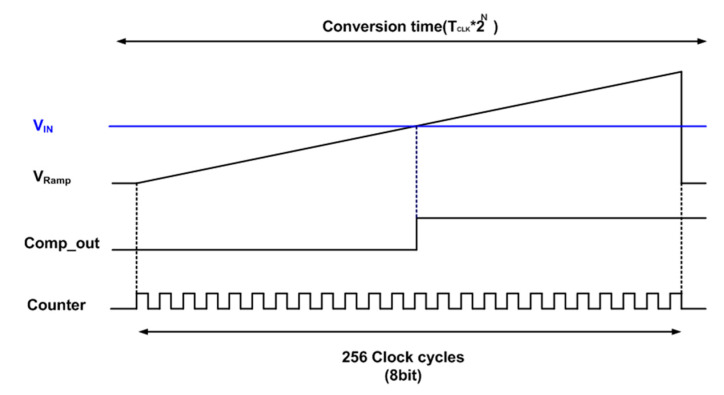
Brief timing diagram of the CIS operation.

The proposed edge-detection CIS was designed and fabricated with a 90-nm 1-poly 5-metal CIS backside illumination process. The supply voltages are 3.3, 2.8, and 1.2 V for analog, pixel, and digital circuit blocks, respectively. The image resolution is 1920 × 1440 pixels, and the ADC resolution is 8 bits for normal images. Figure 6 shows the timing diagram of the 8-bit SS-ADC. The input voltage range of ADC is from 1.5 to 2.2 V. With the pixel voltage (V_IN_), ramping voltage (V_RAMP_) is entered to two inputs of a comparator. When the V_RAMP_ is equal to V_IN_, the output of the comparator is flipped from logic ‘L’ to ‘H’. An 8-bit digital counter starts counting the time that the comparator is flipped from the starting V_RAMP_. For example, when V_IN_ is 2.2 V (the maximum voltage of the ADC input range), the darkest images are obtained, resulting in 0 code. On the other hand, when V_IN_ is 1.5 V, the brightest images are obtained, resulting in the full code of output, 256 codes. It should be noted that the 1 LSB of the ADC in time is 0.98 ns in this paper. Table 1 shows the post-layout simulation results in the process, supply voltage, and temperature variations. The best case is FF corner, 3.63 V analog supply voltage (A_VDD_), and −45 °C temperature, while the worst case is SS corner, 2.97 V A_VDD_, and 135 °C temperature. The post-layout simulation results indicate that the standard deviation of 1 LSB is less than 0.018 LSB in the process, supply voltage, and temperature variations. 

**Figure 7:**
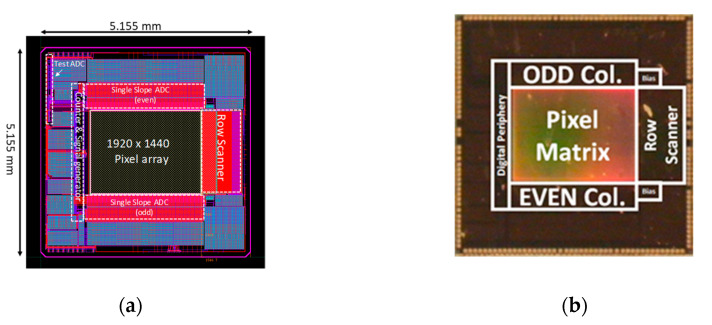
(**a**) Chip layout of the proposed CIS and (**b**) microphotograph of the fabricated CIS.

The layout and a photograph of the proposed CIS are shown in Figure 7a,b, respectively. The column-parallel ADC circuits are located on the top and bottom of the pixel array to secure the layout area of the column-parallel ADC circuits. The pitch of a pixel is 1.4 μm, and the pitch of column-parallel ADC is 2.8 μm. The core size, excluding the I/O pad, is 3.75 mm × 3.75 mm, and the APS array size is 2.68 mm × 2.02 mm. The fill factor is approximately 52.55%, and the total power consumption at 60 fps is 9.4 mW.

### 3.2. Measurement Results

**Figure 8:**
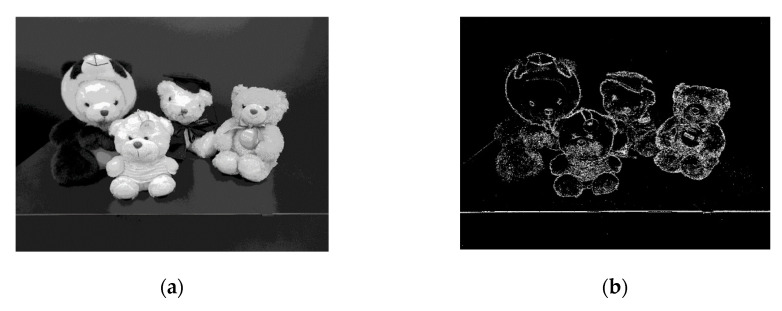
(**a**) An 8-bit black and white image and (**b**) an edge image with the proposed mask circuits.

The full chip was measured by using a field-programmable gate array (FPGA) board to produce the required control signals for the operation of the clocked comparator, memory block, and so on. A signal that was configured through Xilinx was applied to the design circuit through the motherboard by using the FPGA board, and the data were received and measured by the computer. The proposed CIS can capture both normal 8-bit images and edge images, examples of which are shown in Figure 8. 

Table 2 shows the comparison results of Pratt’s figure of merit (PFOM) with other masks, such as Sobel, Roberts, and Prewitt. PFOM [29] is given as

(1) $$R=\frac{1}{I_{N}}\sum_{i=1}^{I_{A}}\frac{1}{1+ad^{2}}$$

where *I_N_* = max{*I_A_*, *I_I_*}, and *I_A_* and *I_I_* are the number of pixels of a reference image and test image, respectively. Since we compared the same image resolution for the reference image and test images, *I_A_* is equal to *I_I_*, which is 2,764,800 (1920 × 1440). *d* is the distance of separation of an actual edge pixel to a line of reference edge pixel, and *a* is the scaling constant (=1/9 following Pratt’s original work). We chose an edge image with the Sobel mask as a reference image and evaluated PFOM. Since a test edge image with the Sobel mask is the same as the reference image with the Sobel mask, PFOM is 100%, while different masks show degradation of PFOM. In the edge image captured by the proposed CIS in edge detection mode (output is 1-bit), PFOM is the lowest (PFOM: 94.96). However, we observed that with a change in the edge threshold from 16 LSB to 8 LSB, the PFOM increased to 95.50%. Since the proposed edge detection circuit is capable of column-wise detection only, the mask is far simpler than others. However, PFOM can be improved with configurable edge thresholds.

**Figure 9:**
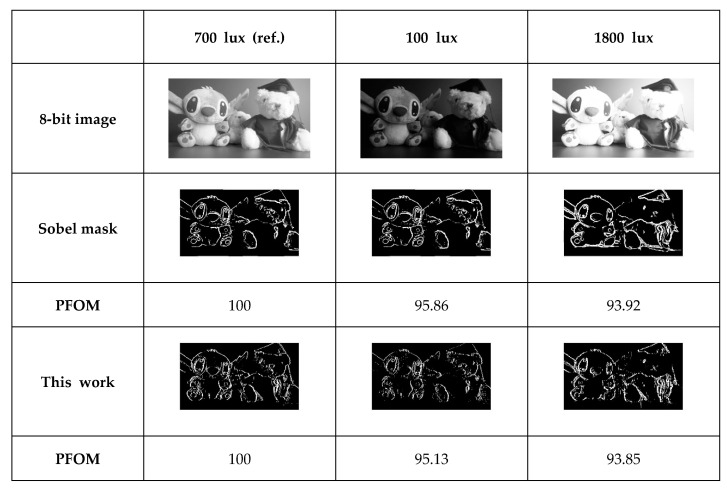
Comparison of edge images between Sobel and the proposed mask with different illuminances.

In addition, we compared the PFOM and edge images of the Sobel mask and the proposed mask with different lux, as shown in Figure 9. We chose the edge image with 700 lux of illuminance as a reference image and calculated PFOM. With different lux, some parts of the test images are saturated in 1800 lux or not distinguishable in 700 lux. Although the proposed mask is capable of column-wise detection only, the degradation trend of PFOM with different illuminances is similar to that of the Sobel mask, which has both column- and row-wise edge detection.

**Figure 10:**
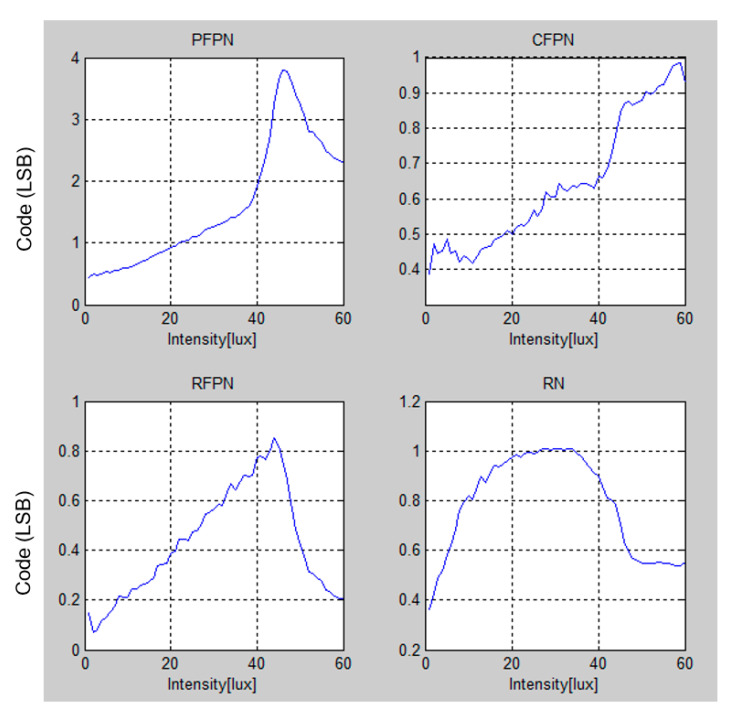
Noise measurement results of the proposed CIS (8-bit images) with different illuminances.

Figure 10 shows the noise performance of the proposed CIS. It should be noted that the ADC of the input range is about 700 mV, and 1 LSB is 2.73 mV. The noises were measured under dark illuminance with 8× of analog gain. When operating as an 8-bit image sensor, pixel fixed pattern noise (PFPN) is within 4 LSB (1.367 mV), and column fixed pattern noise (CFPN), row fixed pattern noise (RFPN), and random noise (RN) were both measured within 1 LSB (342 μV).

Table 3 summarizes the main performance indicators of the proposed edge-detection image sensor. Table 4 compares this sensor’s performance with those of other existing edge-detection image sensor circuits. In [5], a digital circuit that implements the commonly used mask technique is reported. After scanning multiple rows simultaneously and calculating horizontal, vertical, and diagonal edges, the area of the digital circuit was quite large, resulting in low resolution, low conversion speed, and high power consumption. In [6], the analog signal was directly converted to the frequency domain signal at the same time that the mask technique was applied to reduce power consumption and achieve high-speed conversion. Thus, the resolution was quite low, prohibiting its use for high-resolution CIS applications.

In [7], the circuit size was reduced by using the histogram distribution of the image without the mask, but unstable edge images under different illuminations were obtained. In this paper, we propose a built-in mask in CIS without increasing the area compared with that of conventional CISs. In addition, a stable and high-resolution edge image can be obtained regardless of the illumination. Unlike other mask algorithms, the proposed mask algorithm can be applied to any type of column-parallel ADC. Therefore, speed and power consumption can be further improved by using various ADCs.

## 4. Conclusions

In this paper, we present an edge-detection CIS with a proposed built-in mask that was fabricated in a 1-poly 5-metal 90-nm CIS process. We show that the built-in masks for a simple CV function like edge detection can be implemented in column-parallel circuits without an additional ISP. The proposed mask circuit, consisting of an additional XOR and a flip-flop in each column, performs edge detection while maintaining the column-parallel SS-ADC in the conventional CIS structure. We demonstrate that both normal 8-bit images and edge-detection images can be obtained with 9.4 mW of power consumption at 60 fps. Therefore, the proposed built-in mask can apply any column-parallel ADC types, such as successive approximation registers, sigma-delta, etc., to add edge-detection functions. We believe that the proposed edge-detection CIS can be used for low-power computer vision applications in a variety of consumer electronics that have limited power budgets. 

## Figures and Tables

**Table 1:** ΔLSB (=0.985 ns) of an 8-bit ADC under process variation (unit: ns).

Corner	Max.	Min.	Mean	Std. Dev
Best	1.010	0.969	0.980	6.16 × 10^−3^
Nominal	1.010	0.970	0.981	5.17 × 10^−3^
Worst	1.250	0.924	0.983	1.80 × 10^−2^

**Table 2:** Comparison of PFOM of other masks with an image captured by the proposed CIS (8-bit, Figure 8a) and this work with an edge detection sensor (1-bit, Figure 8b).

PFOM (%)	Sobel	Roberts	Prewitt	This Work
Sobel (reference)	100	96.50	99.75	94.96

**Table 3:** Performance summary of the proposed edge-detection CIS.

Array Format	FHD (1920 × 1440)
Pixel Size	1.4 µm × 1.4 µm
ADC Resolution	8-bit
Frame Rate	60 fps
Dynamic Range	61 dB
Power Supply	3.3 V (analog)/2.8 V (pixel)/1.2 V (digital)
Power Consumption	9.4 mW
	90 µW (per column)
	0.4 µW (per column at power shutoff)
Area	26.57 mm^2^ (5.15 mm × 5.15 mm)
Process	90-nm 1P5M BSI CIS

**Table 4:** Performance comparison of the proposed CIS.

	[5]	[6]	[7]	This Work
Edge Image	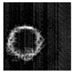	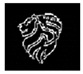	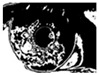	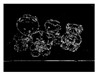
Process	0.18-µm 1P 5M CMOS	0.18-µm 1P 4M CIS	0.18-µm 1P 4M CIS	90-nm 1P 5M CIS
Resolution	70 × 68	105 × 92	174 × 144	1920 × 1440
Pixel Pitch	25.7 µm	8 µm	2.2 µm	1.4 µm
Voltage Supply	1.8 V	1.6 V	3.3 V	3.3 V
Frame/s	28	30	520	60
Power	110 mW	8 mW	2.8 mW (60 fps)	9.4 mW (60 fps)
Fill Factor	17%	11.69%	40%	52.55%
